# Self-Standing Porous Aromatic Framework Electrodes for Efficient Electrochemical Uranium Extraction

**DOI:** 10.1021/acscentsci.3c01291

**Published:** 2023-12-13

**Authors:** Dingyang Chen, Yue Li, Xinyue Zhao, Minsi Shi, Xiaoyuan Shi, Rui Zhao, Guangshan Zhu

**Affiliations:** Key Laboratory of Polyoxometalate and Reticular Material Chemistry of Ministry of Education, Faculty of Chemistry, Northeast Normal University, Changchun 130024, China

## Abstract

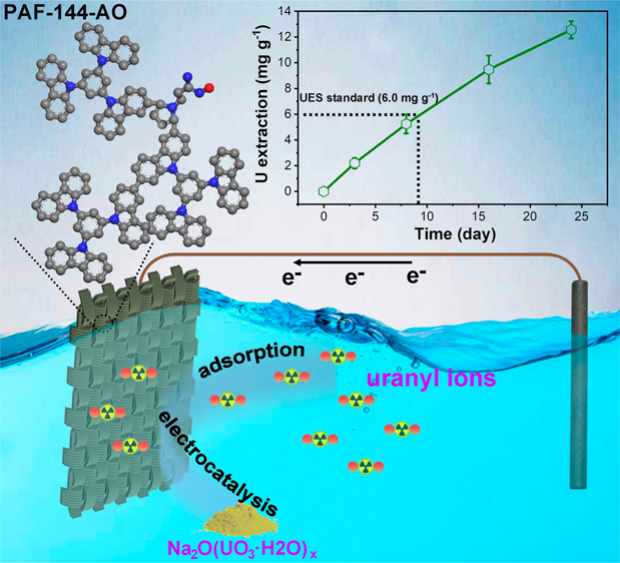

Electrochemical uranium extraction from seawater provides a new opportunity for a sustainable supply of nuclear fuel. However, there is still room for studying flexible electrode materials in this field. Herein, we construct amidoxime group modified porous aromatic frameworks (PAF-144-AO) on flexible carbon cloths *in situ* using an easy to scale-up electropolymerization method followed by postdecoration to fabricate the self-standing, binder-free, metal-free electrodes (PAF-E). Based on the architectural design, adsorption sites (amidoxime groups) and catalytic sites (carbazole groups) are integrated into PAF-144-AO. Under the action of an alternating electric field, uranyl ions are selectively captured by PAN-E and subsequently transformed into Na_2_O(UO_3_·H_2_O)_*x*_ precipitates in the presence of Na^+^ via reversible electron transfer, with an extraction capacity of 12.6 mg g^–1^ over 24 days from natural seawater. This adsorption–electrocatalysis mechanism is also demonstrated at the molecular level by *ex situ* spectroscopy. Our work offers an effective approach to designing flexible porous organic polymer electrodes, which hold great potential in the field of electrochemical uranium extraction from seawater.

## Abstract 2

Self-standing, binder-free, metal-free porous aromatic framework-based electrodes were prepared and used for electrochemical uranium extraction.

## Introduction

Nuclear energy is a low-carbon energy source to displace fossil fuels and provides an important guarantee for the green development of the economy and environment.^1,2^ Uranium (U) is the main fuel of nuclear power reactors; however, the limited uranium resource reserves on land have become a serious obstacle to sustainable nuclear energy industry development. Comfortingly, the uranium reserves in seawater are estimated to be 4.5 billion tons, nearly 1000 times larger than terrestrial uranium reserves.^3,4^ As a result, uranium extraction from seawater (UES) has been widely investigated, and much attention has been paid to this field. However, UES is still a hugely challenging task owing to the extremely low U concentration (3.3 ppb), abundant interfering ions, and complex environment in seawater.^5−8^ The development of uranium extraction materials and methods with high capacity, fast kinetics, and good selectivity is of great significance.

Recently, electrochemical uranium extraction, an emerging and attractive method, has received more and more attention.^9^ In the report by Cui’s group, a half-wave rectified alternating current electrochemical (HW-ACE) method was applied to extract uranium using the amidoxime-functionalized carbon electrode.^10^ During this electrochemical process, adsorbed uranyl ions (UO_2_^2+^) were reduced to insoluble UO_2_ that was deposited on the electrode. Compared with traditional physicochemical adsorption methods, the electrochemical method could significantly enhance the uranium extraction capacity and rate. Inspired by this work, Wang and co-workers proposed the adsorption–electrocatalysis system for uranium extraction using an amidoxime-functionalized metal–nitrogen–carbon (M–N_*x*_–C–R) catalyst.^4,11^ The absorbed UO_2_^2+^ was converted to uranium precipitates via electrocatalysis, and a high uranium extraction capacity of 1.2 mg g^–1^ day^–1^ was achieved. Though the electrochemical uranium extraction method has been well established, the developed electrode materials still have some drawbacks. Conventional functional electrode materials possess a low specific surface area, leading to limited available active sites.^12^ M–N_*x*_–C–R electrocatalysts need to mix with binders and conductive agents to prepare the electrodes.^4,11^ This process is complicated, and the active materials easily fall off after multiple processing steps. Therefore, it is urgent to design self-standing porous electrodes integrating adsorption sites and catalysis sites for electrochemical uranium extraction.

Porous organic polymers (POPs) are constructed from organic building blocks via the covalent bonds to form the stable pore frameworks.^13,14^ The common POPs are porous aromatic frameworks (PAFs), covalent organic frameworks (COFs), conjugated microporous polymers (CMPs), etc., and they feature high surface area, good stability, and easy modification.^15,16^ Recently, some POPs have been investigated for electrochemical uranium extraction, and excellent extraction performances were obtained.^3,17,18^ However, they usually exist in powder form and do not easily form a macroscopic shape. They needed the above-mentioned slurry coating method to obtain the electrodes. Moreover, their synthesis processes require harsh experimental conditions. Recently, it has been reported that electropolymerization is an easy and effective strategy to bond the electroactive monomers to POPs, which are deposited on the conductive substrates.^19,20^ This electropolymerization process is green, catalyst-free, and time-saving. In addition, regulating the chemical structures of the monomers could endow the electropolymerized POPs with adsorption sites and catalysis sites.^21,22^ Thus, the electropolymerization of designed POPs on suitable flexible and conductive substrates is a good candidate for self-standing porous electrodes in electrochemical uranium extraction technology.

Herein, the self-standing porous aromatic framework electrodes were fabricated through facile electropolymerization and postdecoration of amidoxime groups. Owing to their adsorption sites, catalytic sites, abundant mass transfer channels, and electric field assistance, the obtained PAF electrodes could effectively realize electrochemical uranium extraction via adsorption–electrocatalysis processes. This electrochemical process showed the kinetics to be 3-fold faster than the physicochemical adsorption and ultrahigh removal capacity at low equilibrium concentration (1413.9 mg g^–1^ at 4.6 mg L^–1^) from uranium-spiked seawater. Moreover, they also exhibited satisfying selectivity against common competing ions. In the natural seawater test, a high uranium extraction capacity of 12.6 mg g^–1^ was achieved after 24 days of operation. Additionally, we analyzed in detail the electrochemical uranium extraction mechanism using our PAF electrodes and clarified the species transformation during the adsorption–catalysis process, offering great potential for large-scale application.

## Results and Discussion

### Preparation and Characterization of PAF Electrodes

**Figure 1:**
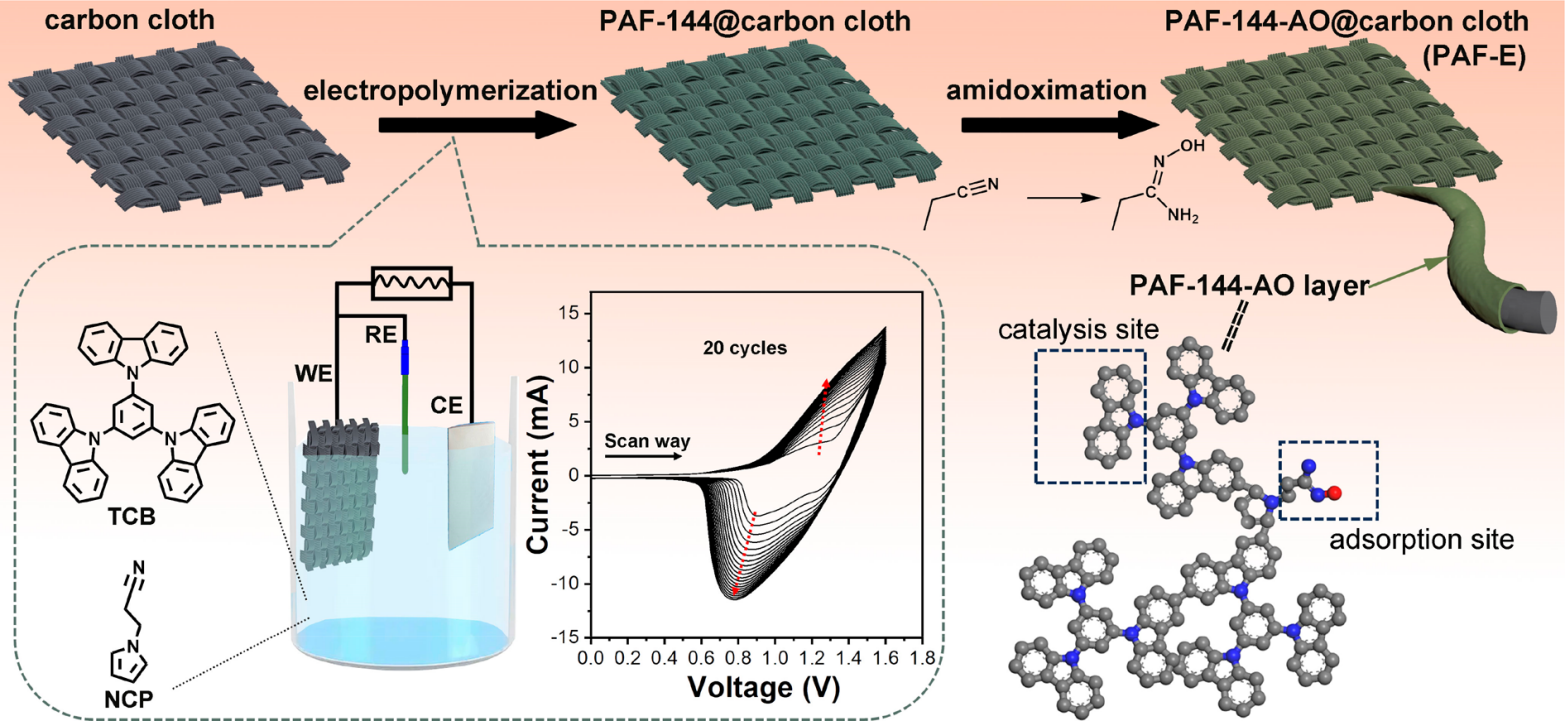
Schematic representation for the fabrication of self-standing porous aromatic framework electrodes. Inset: Electropolymerization setup and CV profiles.

The preparation of our targeted electrodes is schematically described in Figure 1. To endow the PAF materials with a self-standing property, textile carbon cloths served as the substrates on which the PAFs could be electropolymerized onto the carbon fibers conformally. Two electroactive monomers, 1,3,5-tris(*N*-carbazolyl)benzene (TCB) and *N*-(2-cyanoethyl)pyrrole (NCP), were chosen as the building units to co-construct the PAFs (PAF-144). Rigid TCB has three reactive carbazole blocks that could form 3D porous networks. The cyano groups in NCP could be modified into amidoxime groups, which were the effective electroadsorption sites for uranyl ion binding. Moreover, the electroactive sites with redox properties on the PAFs could act as the electrocatalysis sites to convert the adsorbed uranyl ions. The electropolymerization (EP) process was performed using cyclic voltammetry (CV) ranging from 0.0 to 1.6 V (vs Ag/Ag^+^) (Figure S1). The positive scans corresponded to the oxidation of carbazole and pyrrole to cationic radicals. The generated cationic radicals coupled with each other to form the dimeric species, which could be easily oxidized to cation radicals. During the negative scans, the cations were reduced to their neutral state (Figure S2). It was noted that both oxidation and reduction peak currents rose gradually with the increasing number of CV cycles, indicating the polymerization growth of PAF-144 on the carbon cloths. After the EP, the PAF-144@carbon cloth composites were reacted with hydroxylamine hydrochloride to accomplish the conversion of cyano groups to amidoxime (AO) groups to create PAF-144-AO, finally yielding the flexible porous aromatic framework electrode (PAF-E) (Figure S3). Moreover, this method was versatile, and PAF-144-AO could be deposited on other conductive substrates.

**Figure 2:**
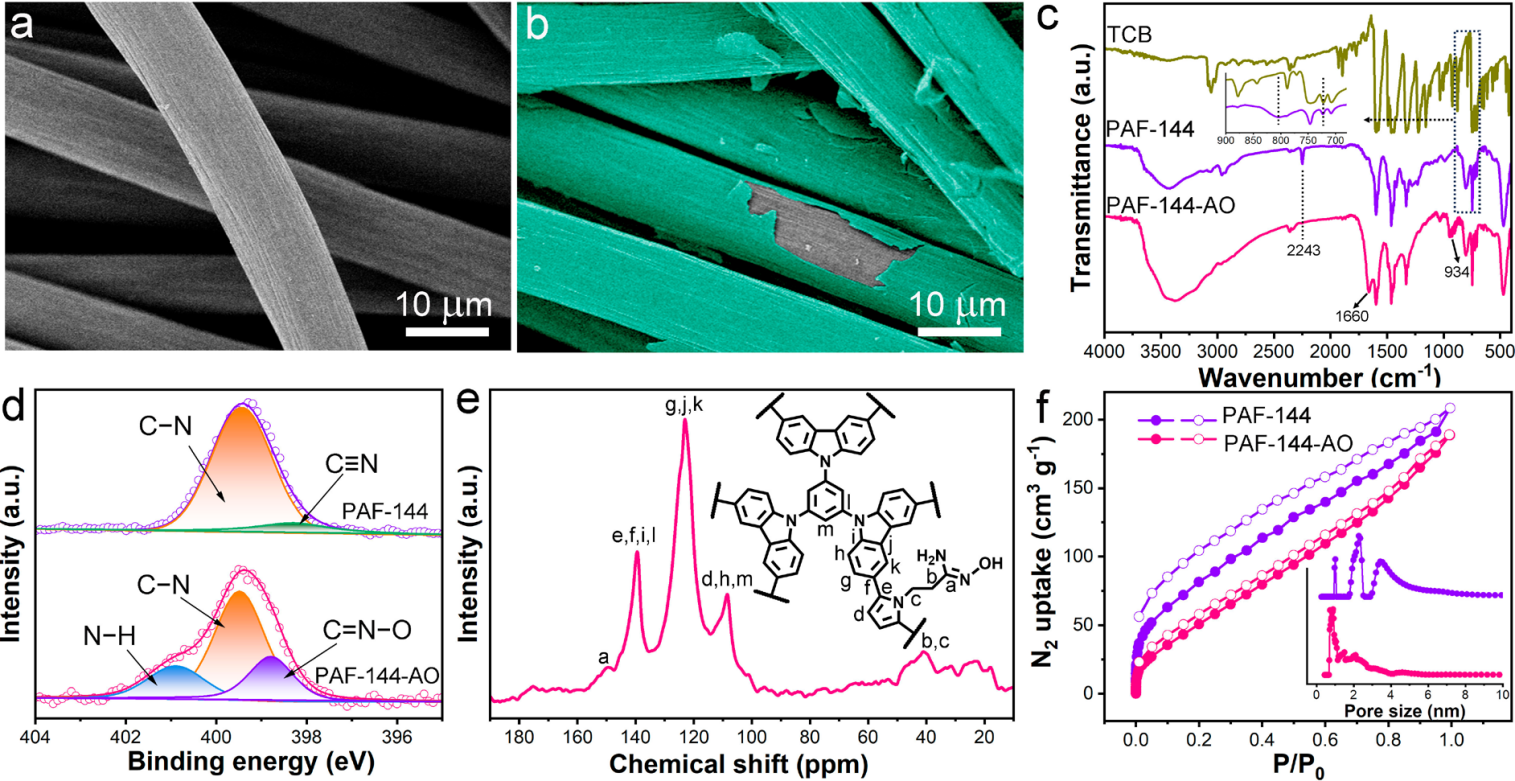
Characterization of the obtained materials. SEM images of (a) carbon cloth and (b) PAF-E. (c) FT-IR spectra of the monomer and the PAFs. (d) High-resolution N 1s spectra of the obtained PAFs. (e) Solid-state ^13^C CP/MAS NMR spectrum of PAF-144-AO. (f) N_2_ adsorption–desorption isotherms of PAF-144 and PAF-144-AO (the inset is their pore size distributions).

The scanning electron microscopy (SEM) images with low magnification showed that electropolymerization and amidoximation reactions did not destroy the macroscopic feature of the carbon cloth (Figure S4). In the high-magnification images of carbon cloth and PAF-E (Figure 2a and 2b), we observed that the obvious and dense PAF-144-AO layer was coated on the carbon fibers uniformly, suggesting the feasibility of the EP process to grow PAFs onto the conductive substrates. FT-IR spectroscopy was used to identify the chemical structures of the obtained PAFs (Figure 2c). Compared with the monomer TCB, a new peak at 803 cm^–1^ appeared in the spectrum of PAF-144, which was assigned to the trisubstituted carbazole, and the peak at 721 cm^–1^ belonging to the bisubstituted carbazole became relatively weakened, suggesting that most of the carbazole sites had been linked to form the networks.^21^ Moreover, the absorption band at 2243 cm^–1^ was attributed to the characteristic peak of C≡N (Figure S5),^23^ suggesting that NCP had been polymerized and incorporated into the porous networks. In the spectrum of PAF-144-AO, the peak belonging to C≡N almost disappeared, and new peaks could be observed at 1660 and 934 cm^–1^, corresponding to the C=N bond and the N–O bond, respectively. This phenomenon confirmed that cyano groups were successfully transformed into amidoxime groups.^24,25^ The element content changes of C, N, and O for the obtained materials also corresponded to PAF-144 growth and amidoxime modification (Figure S6). The amidoxime modification was further evidenced by X-ray photoelectron spectroscopy (XPS, Figure S7). The high-resolution N 1s spectra of PAF-144 could be divided into two peaks representing the C–N bond (399.5 eV) and the C≡N bond (398.3 eV) (Figure 2d).^26^ However, PAF-144-AO showed three fitted peaks, and the new two peaks at 400.9 and 398.8 eV were assigned to N–H and C=N–O in the amidoxime groups, respectively, suggesting the modification process.^26,27^ PAF-144-AO was also evaluated by solid-state ^13^C nuclear magnetic resonance (^13^C NMR) spectroscopy (Figure 2e), which identified its chemical structures well and agreed with the FT-IR and XPS results. Due to the hydrophilicity of amidoxime ligands,^28^ the water contact angle of the PAF-144-AO electrode decreased to 47.0° from the 128.6° of the PAF-144 electrode (Figure S8). The good hydrophilicity was beneficial to the diffusion of uranyl ions in the electrodes, thus enhancing U capture. The pore properties of the PAFs were also analyzed by the nitrogen adsorption–desorption isotherms (Figure 2f). The Brunauer–Emmett–Teller (BET) surface areas for PAF-144 and PAF-144-AO were 233 and 125 m^2^ g^–1^, respectively. Moreover, the pore sizes underwent clear shrinkage after the amidoximation process. This was because the introduction of amidoxime groups could increase the mass and lead to pore filling. The dominant pores of PAF-144-AO were located at 0.87, 1.54, and 2.20 nm, respectively, which were beneficial to the diffusion of uranyl ions (a maximum length of 0.60–0.68 nm).^18,29^ The conductivity of the electrodes was studied by electrochemical impedance spectroscopy (EIS) (Figure S9). After PAF-144-AO was coated, the semicircle radius did not show a significant change in comparison to pure carbon cloth. Owing to the conjugated structures in PAF-144-AO and good electrical conductivity of the carbon cloth substrate, the charge transfer in PAF-E was low, suggesting its potential in electrochemical applications.

### Electrochemical Uranium Extraction from Spiked and Natural Seawater

**Figure 3:**
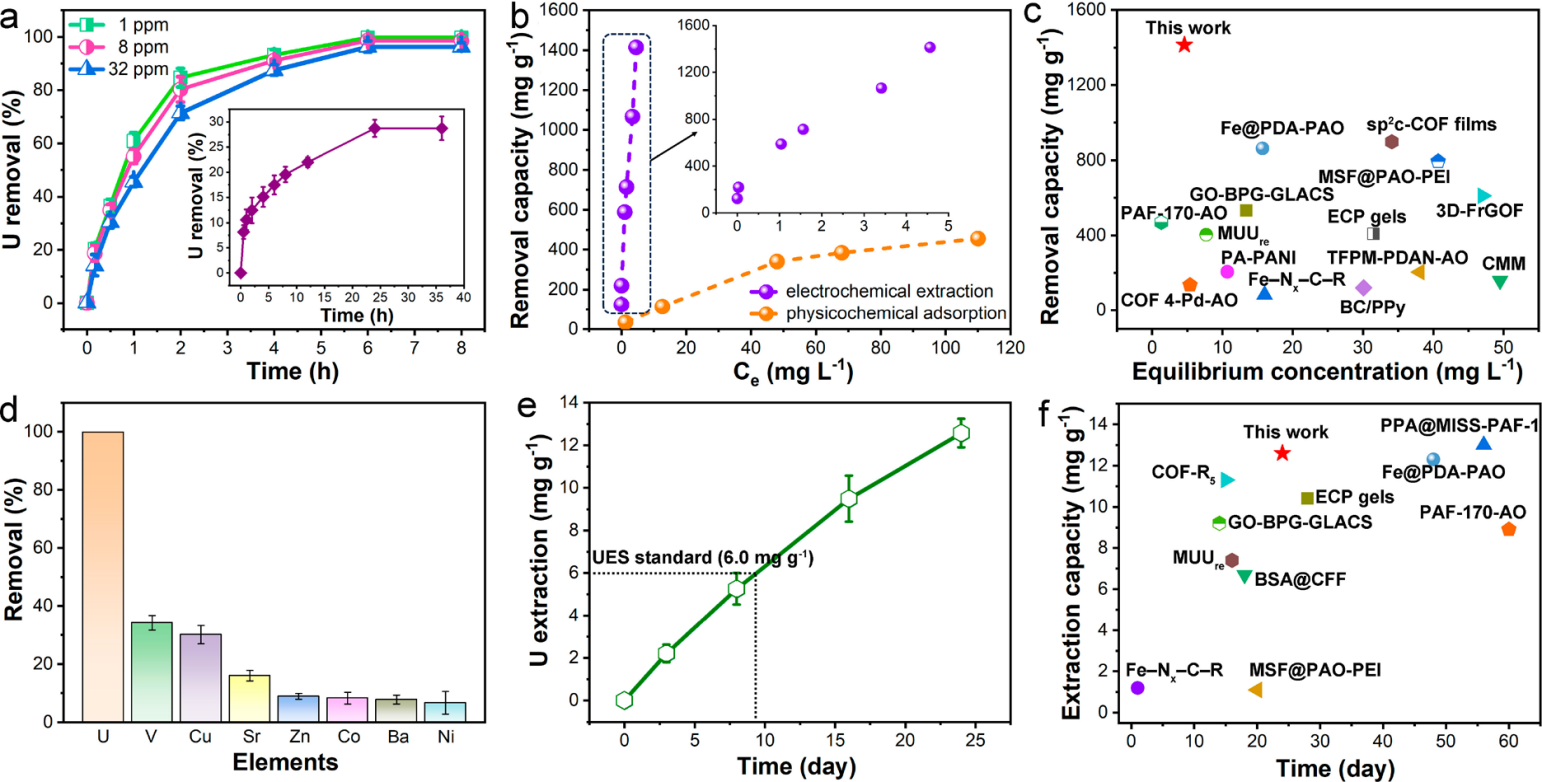
Electrochemical uranium extraction performance by PAF-E. (a) Extraction kinetics under different initial U concentrations (inset is the kinetics from the physicochemical adsorption). (b) Effect of U concentration on U removal for electrochemical extraction and physicochemical adsorption. (c) Comparison of the U removal capacities at low equilibrium concentrations with other U extraction materials (corresponding references are presented in Table S1). (d) U removal by PAF-E in the presence of various interfering ions (the concentrations of U and the interfering ions are equal to 10 ppm). (e) U extraction ability from natural seawater. (f) Comparison of the U extraction capacities in natural seawater (corresponding references are presented in Table S1).

Based on the above characterization, we further investigated the electrochemical extraction of uranium using the HW-ACE method with a frequency of 400 Hz from U-spiked seawater, in which PAF-E was utilized as the negative electrode and a graphite rod was the positive electrode (Figure S10). The effect of the applied voltage on uranium removal was also investigated (Figure S11). Based on the results, an alternating voltage of between −5 and 0 V was applied in this work. The electrochemical extraction could reach equilibrium within 8 h at the tested U concentrations (1, 8, and 32 ppm), and the U removal efficiencies all exceeded 96.0% (Figure 3a). For the physicochemical adsorption (initial U concentration = 32 ppm), the equilibrium time needed was 24 h, and the removal efficiency was only 28.7% (inset in Figure 3a). Moreover, the residual concentration of the electrochemical U removal from the initial concentration of 1 ppm was only 13.2 ppb, suggesting its good trace removal ability.^30^ In the uranium extraction experiments under different initial concentrations, the maximum adsorption capacity from physicochemical adsorption was 483.2 mg g^–1^ (Figure 3b). In sharp contrast, the uranium extraction by the electrochemical method seemed to show no saturation. At an equilibrium concentration of 4.6 mg L^–1^, the U removal capacity could reach 1413.9 mg g^–1^, which surpassed most of the reported U extraction materials with different methods (Figure 3c and Table S1). We also tested the electrochemical U removal under the high initial U concentration of 500 ppm. The removal capacity was 7450.9 mg g^–1^, and this value was better than most of the materials reported so far (Table S1). The electrochemical uranium extraction capacity with pure carbon cloth was also conducted (Figure S12). The removal capacity of pure carbon cloth was only 3.7 mg g^–1^, which was 0.26% of the capacity of PAF-E, suggesting that the uranium extraction capacity of PAF-E was almost attributed to the PAF material. To demonstrate the effect of porous structure and amidoxime groups, the electrodes from the individual electropolymerization of TCB or NCP were prepared, and the NCP polymerized electrodes also underwent the amidoximation reaction. (The synthesis procedure is shown in the Supporting Information.) In addition, PAF-144@carbon cloth composites were also included in this comparison. By contrast, the electrochemical U extraction capacities of these electrodes were much lower than that of PAF-E under the same conditions (Figure S12), suggesting that the porous networks and amidoxime groups contributed greatly to the electrochemical U extraction. The existence of amidoxime groups in the PAF-E offered effective chelation sites for uranyl ion binding during the electrochemical process. The porous networks could provide abundant available adsorption and catalysis sites for the uranyl ion capture and conversion. Selectivity is an important factor in the uranium extraction from seawater. As shown in Figure 3d, the U removal efficiency was much higher than those of other metal ions. This good selectivity was attributed to the alternating voltage applied to the electrodes, which repelled unbound ions (Figure S10). The stability of PAF-E was evaluated by the extraction cycles (Figure S13). After 10 cycles, the removal efficiency was still greater than 98.0%. Encouraged by the aforementioned U removal results, we conducted the uranium extraction experiments from natural seawater (U concentration ≈ 3.3 ppb) (Figure S14). When prolonging the operation days, the U extraction capacity increased gradually. The performance could reach the uranium extraction standard (6.0 mg g^–1^) on day 9. On day 24, the extraction capacity was 12.6 mg g^–1^ and the saturation was still not reached (Figure 3e). This capacity was superior to those of most of the reported uranium extraction materials (Figure 3f and Table S1). The obtained uranium extraction performance indicated that PAF-E showed great potential in the uranium extraction from seawater.

### Uranium Extraction Mechanism Analysis

**Figure 4:**
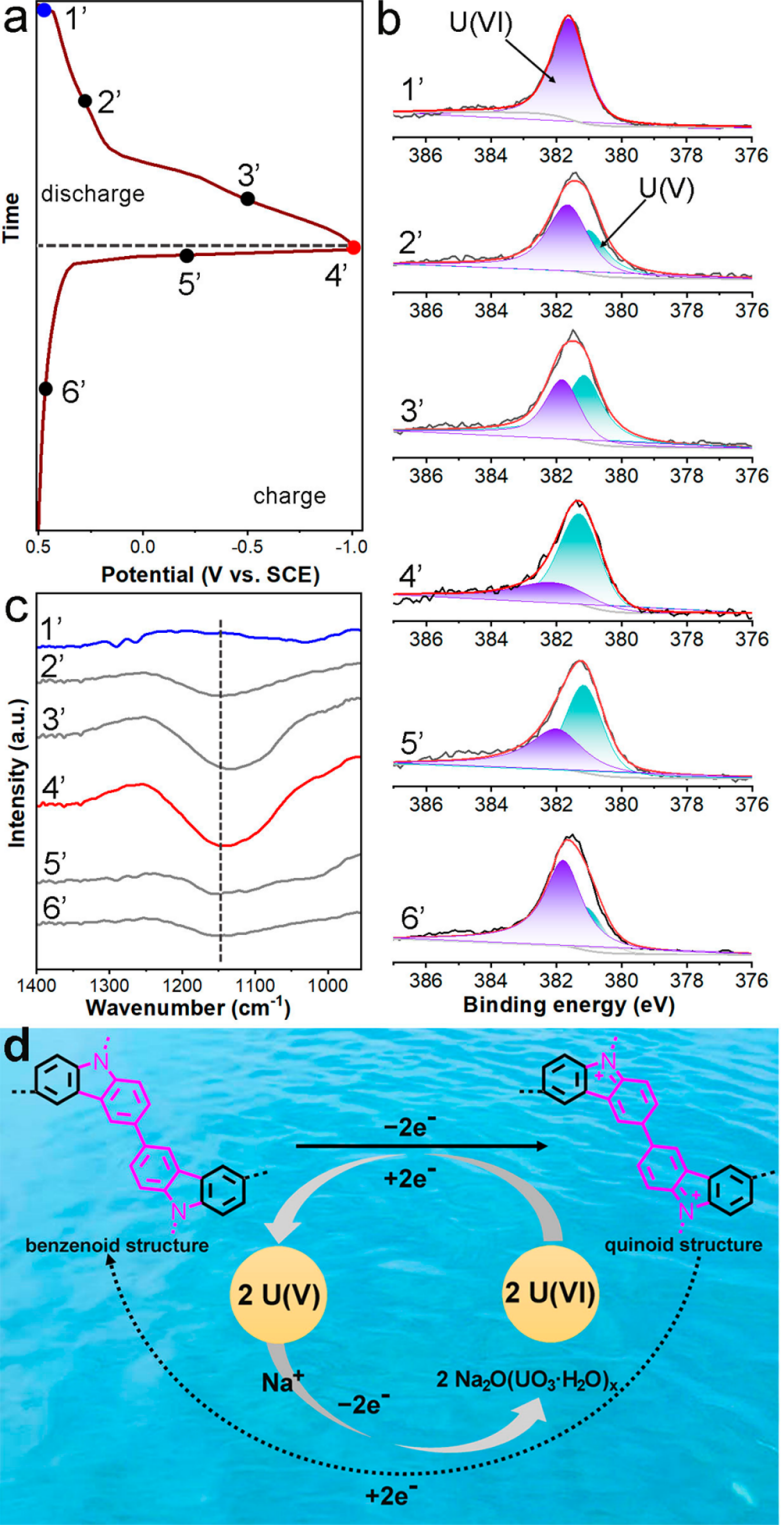
Mechanism analysis. (a) GCD profile of PAF-E in the U solution. (b) *Ex situ* XPS results of high-resolution U 4f spectra and (c) *ex situ* FT-IR spectra of PAF-E at the marked points in the GCD process. (d) Schematic diagram of the electrochemical uranium extraction by the PAF-E.

We further investigated the electrochemical extraction mechanism through instrumental characterizations. The survey XPS spectrum of PAF-E after the uranium extraction showed obvious U 4f peaks at 380–390 eV (Figure S15a), and the high-resolution O 1s spectra after the uranium extraction showed a new peak at 530.9 eV belonging to O–U (Figure S15b and S15c),^31^ indicating that U species were adsorbed by the amidoxime groups in PAF-144-AO during the electrochemical extraction. Cyclic voltammetry (CV) tests were also performed for the PAF-E using the three-electrode electrochemical cell in natural seawater and uranyl-spiked seawater (Figure S16). Compared with the CV curve in natural seawater, there were obvious oxidation and reduction peaks in uranyl-spiked seawater. The peak at −0.26 V was attributed to the reduction of U(VI) to U(V), and the peak at 0.08 V belonged to the oxidation of U(V) to U(VI).^4^ In addition, during the electrochemical extraction process at the high U concentration (500 ppm), we could observe obvious yellow flocs around the PAF-E electrodes, and their amount increased gradually until the extraction equilibrium (Figure S17). If we dropped the voltage, the yellow flocs not bound tightly would fall off of the electrodes. According to the XRD and EDS results (Figure S18), the collected yellow precipitates mainly contained Na_2_O(UO_3_·H_2_O)_*x*_, which agreed with the previous reports.^11,17^ These results suggested that the electrocatalysis process was also involved in electrochemical uranium extraction. After the electrochemical extraction, there were still obvious precipitates on the electrodes (Figure S19), and the EDS element mapping verified their uniform adhesion on the electrode surfaces. This meant that the PAF-E electrodes had the ability to immobilize U species, especially at low U concentration. After the uranium extraction from natural seawater (related to Figure 3e), yellow powders could be observed (Figure S20a). According to the EDS element, the U element was detected in the spectrum (Figure S20b), indicating the effective uranium extraction performance from natural seawater using our electrochemical system. Furthermore, the control experiment was performed by investigating the electrochemical uranium removal in a U solution prepared from deionized water. No solid precipitates were formed under these conditions (Figure S21), indicating the necessity of sodium ions for the formation of Na_2_O(UO_3_·H_2_O)_*x*_ precipitates in the seawater system. To explain the electrocatalysis reaction between PAF-E and uranyl ions, galvanostatic charge–discharge (GCD) profiles were obtained in the three-electrode system (Figure 4a). The *ex situ* XPS was used to monitor the dynamic evolution of U valences (Figure 4b).^9^ In the discharging process, the content of U(VI) gradually decreased and the content of U(V) gradually increased. This phenomenon was mainly ascribed to the reduction of uranyl to the U(V) intermediate. Afterward, the content of U(V) showed a decreasing trend, and the content of U(VI) increased when the electrode was recharged to 0.5 V, meaning that the electron transfer occurred between PAF-E and U species. Carbazoles were the electroactive groups that could act as the redox sites in the electrocatalysis. The gain or loss of electrons could lead to the carbazole transformation between the benzenoid structure and the quinoid structure.^32,33^ Further *ex situ* FT-IR spectra at different discharge/charge states were recorded to confirm the evolution of these structures (Figure 4c). After the reduction of U(VI) in the discharging process, the characteristic peak of the electronic-like absorption of the quinoid ring appeared at 1100 cm^–1^^34^ and its intensity increased gradually. On the contrary, this characteristic peak gradually weakened during the oxidation of U(V), revealing the participation of carbazole groups in the U redox and the existence of a reversible electron-transfer process. Thus, we could conclude that the electrochemical uranium extraction by PAF-E was interpreted as follows: (I) based on the electric polarization effect and the chelation of amidoxime groups, uranyl ions were adsorbed on the PAF-E; (II) then the carbazole groups in PAF-144-AO reduced the U(VI)O_2_^2+^ to U(V)O_2_^+^ via the electron transfer. The generated U(V) was again oxidated to U(VI) in the presence of Na^+^, which accompanied the proton release, ultimately forming Na_2_O(UO_3_·H_2_O)_*x*_ precipitates (Figure 4d).

## Conclusions

We demonstrated facile electropolymerization and the following functionalization to grow PAF-144-AO on carbon cloths, assembling the self-standing and binder-free electrodes for electrochemical uranium extraction. According to the comparative experiments, amidoxime groups, electroactive sites, and porous frameworks synergistically improved the uranium extraction performance via the adsorption–electrocatalysis. This electrochemical process showed higher uptake and faster kinetics in comparison to physicochemical adsorption. The uranium extraction capacity of 12.6 mg g^–1^ could be achieved in natural seawater over 24 days. Importantly, we determined the uranium adsorption–catalysis sites and investigated the transformation of uranium species, giving an in-depth mechanistic understanding of the electrochemical uranium extraction by our PAF-E. This work laid a platform for the design of self-standing porous organic polymer electrodes and provided an effective strategy for the uranium extraction from seawater through the electrochemical process.

## Supporting Information Available

Other electrode preparation and uranium extraction experiments, CV curves, electropolymerization mechanism, optical image, and SEM image of electrodes; FT-IR spectrum of NCP; SEM-EDS patterns; survey XPS spectra; water contact angles; impedance Nyquist plots; description of the HW-ACE method; effect of the applied voltage; comparison of the uranium removal performance; uranium electrochemical removal by different electrodes; durability performance; device used in electrochemical uranium extraction; XPS spectra, CV profiles; formation process of yellow flocs; XRD, EDS, and elemental mapping pattern of the collected yellow precipitates; and photograph of uranium extraction in U solution from deionized water (PDF)
