# Supplementary material for: Self-Standing Porous Aromatic Framework Electrodes for Efficient Electrochemical Uranium Extraction
Source: ACS Cent Sci. 2023 Dec 13;9(12):2326–32. doi: 10.1021/acscentsci.3c01291 (PMC10755849; doi:10.1021/acscentsci.3c01291)
Supplement: Supplementary file 1 — oc3c01291_si_001.pdf [file oc3c01291_si_001.pdf]

Supporting Information

Self-standing Porous Aromatic Framework Electrodes for Efficient Electrochemical Uranium Extraction

Dingyang Chen[#] Yue Li[#] Xinyue Zhao, Minsi Shi, Xiaoyuan Shi, Rui Zhao, and Guangshan Zhu**

Key Laboratory of Polyoxometalate and Reticular Material Chemistry of Ministry of Education, Faculty of Chemistry, Northeast Normal University, Changchun 130024, China

E-mail: zhaor814@nenu.edu.cn; zhugs@nenu.edu.cn

Chemicals

All reagents and solvents were purchased from commercial suppliers and used without further purification, unless otherwise noted. The real seawater was collected from the Bohai Sea near the east coast of Qingdao city, China. The real seawater was vacuum filtered through a 0.2-μm filter to remove the large particles and microorganisms before use. Spiked uranium solutions were prepared by dissolving uranyl nitrate into real seawater and the pH of the uranyl−spiked seawater solution was adjusted to 6.0.

Fabrication of the Self-standing PAF based Electrodes

Carbon cloths were first cleaned by sonication in ethanol for 60 min and dried in 80 ℃ vacuum oven, later cut into square shapes as electrode substrates. 1,3,5-tris(N-carbazolyl)benzene (TCB, 50.0 mg) and tetrabutylammonium hexafluorophosphate ($C_{16}H_{36}F_6NP$, 1.5 g) were weighted into a 50.0 mL glass bottle, followed by adding N-(2-cyanoethyl)pyrrole (NCP, 10.5 mg), acetonitrile (12.0 mL) and dichloromethane (28.0 mL), then stirred to form a homogeneous electrolyte solution. A conventional three-electrode cell was used with a carbon rod as the counter electrode, a non-aqueous Ag/Ag^+ organic electrode (CH_2Cl_2) as reference electrode and the treated carbon cloth as the working electrode. Cyclic voltammetry (CV) measurements were conducted in this standard three-electrode system under the voltage range from -1.6 V to 0.0 V for PAF growth. The scan rate was 10 mV s^{-1} and the PAF coated electrode was obtained after 20 cycles. For the amidoximation procedure, the PAF coated carbon cloth was treated in a mixture of hydroxylamine hydrochloride ($NH_2OH{\cdot}HCl$, 250.0 mg), triethylamine (TEA, 0.6 mL), and anhydrous ethanol (10.0 mL) at 70 ℃ for 72 h,

then cleaned with ethanol three times to give the self-standing PAF based electrodes (Scheme 1).

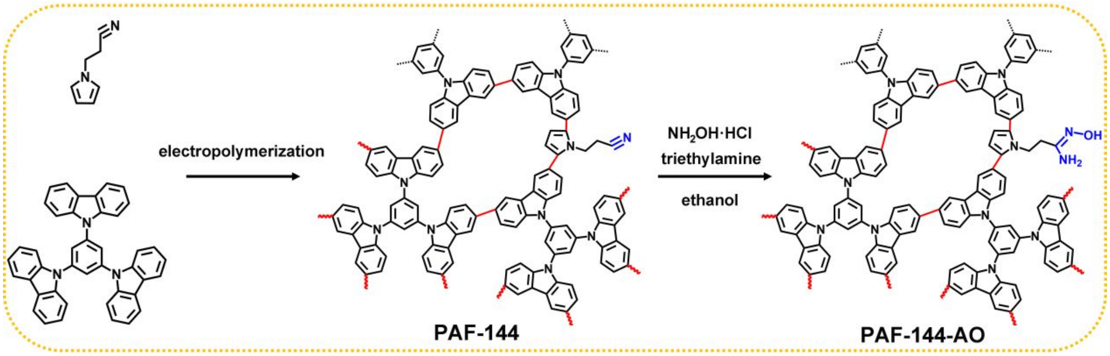

Scheme 1. Synthesis routes and the relevant chemical structures of PAF-144 and PAF-144-AO.

For the comparison, the electrodes from the individual electropolymerization of TCB (Scheme 2) or NCP were prepared and the NCP polymerized electrodes also underwent the amidoximation reaction. The preparation conditions were similar to those of the self-standing PAF based electrodes (Scheme 3).

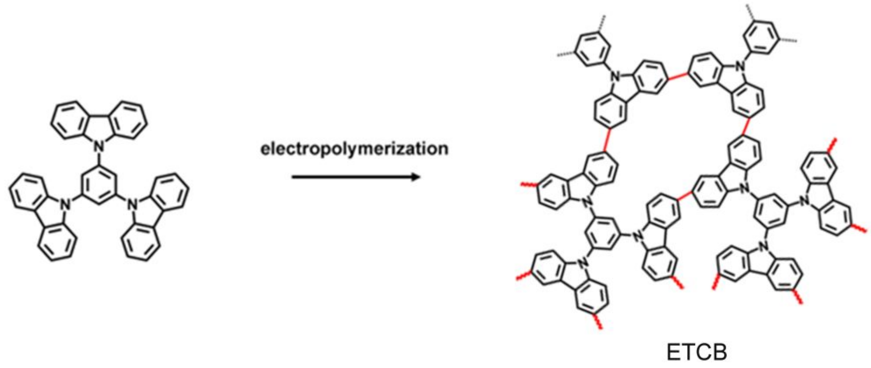

Scheme 2. Synthesis routes and the relevant chemical structures of ETCB.

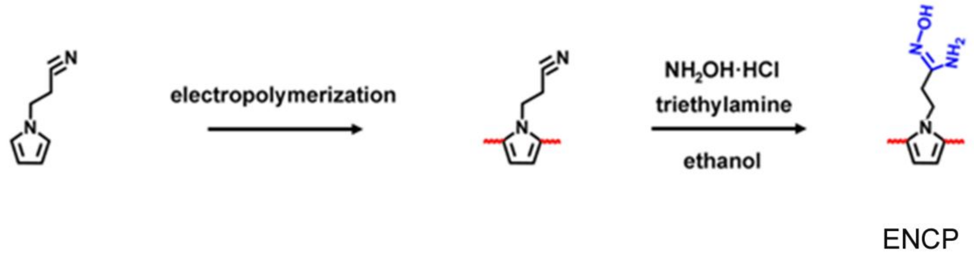

Scheme 3. Synthesis routes and the relevant chemical structures of ENCP.

Characterizations

Field emission scanning electron microscopy (SEM, Shimadzu SSX-550) with Energy-dispersive X-ray spectroscopy (EDS) was used to observe the materials. The Nicolet iS50 Fourier transform infrared spectrometer was applied to record the Fourier-transform infrared (FTIR) spectra of the samples. X-ray photoelectron spectroscopy (XPS) measurements were performed on a Thermo ESCALAB 250 spectrometer with a Mg-Ka (1253.6 eV) achromatic X-ray source. X-ray diffraction (XRD) patterns were tested on a Rigaku SmartLab X-ray diffractometer with a Cu-Kα radiation of λ = 1.5418 Å (40 kV, 30 mA). N_2 adsorption−desorption isotherms were analyzed by a Quantachrome Autosorb iQ SN analyzer at −196 °C. Solid-state ^{13}C cross-polarization magic angle spinning nuclear magnetic resonance (CP/MAS NMR) measurement was performed on a Bruker Avance III model 400 MHz NMR spectrometer at a MAS rate of 5 kHz. Contact angle goniometer (KRüSS DSA 30) was used to measure the contact angles of the fibers. The concentration of uranium was determined by inductively coupled plasma mass spectrometer (ICP-MS) and UV–visible spectrophotometer using Arsenazo III assay. Electrochemical tests were conducted by an electrochemical workstation (Vertex.One, Ivium, Netherlands) with a standard three-electrode system.

Physicochemical Adsorption Experiments

To investigate the physicochemical adsorption performance, PAF based electrodes was immersed into uranyl−spiked seawater solution with stirring. At specific time intervals, the uranium concentration in the solution was analyzed. The adsorption kinetics were performed at an initial concentration of 32 ppm. The isotherm experiments were conducted under certain initial concentrations of 5~120 ppm.

Data Analysis

The removal rate (R%) and the adsorption capacity (q, mg g^{-1}) were calculated through the following equations:

$$R\ (\%) = \frac{(C_0 - C_e)}{C_0} \times 100\% \qquad (1)$$

$$q\ (mg\ g^{-1}) = \frac{(C_0 - C_e)V}{W} \qquad (2)$$

where C_0 and C_e (mg L^{-1}) are the initial and the equilibrium concentration of uranium in the aqueous solution, respectively. V (L) is the volume of the solution, and m (g) is the mass of the PAF material in the electrodes.

Electrochemical Uranium Removal

All electrochemical uranium adsorption experiments were conducted in a standard two−electrode system using a graphite rod as the anode and self-standing PAF based electrode as the cathode. Experiments were conducted over the voltage range from 0 V to −5 V using a frequency of 400 Hz during the tests. The pH of the uranyl−spiked seawater was adjusted to 5.0. The concentrations of the tested uranyl−spiked seawater solution were determined at specific time intervals.

For the selectivity experiments, the electrochemical uranium removal was performed in 10 ppm U-spiked real seawater solutions containing different interfering ions. The interfering ions included 10 ppm VO_3^-, Cu^{2+}, Sr^{2+}, Zn^{2+}, Co^{2+}, Ba^{2+} and Ni^{2+}, which were much higher than their actual concentrations. After the electrochemical removal, the concentrations of different metal ions were recorded.

For the reusability assay, fresh 10 ppm uranium spiked real seawater was used to run the electrochemical uranium removal. After one removal process, the U-loaded electrode was treated by eluting the bound uranium with 0.1 M HNO_3 and the blending solution of 1.0 M Na_2CO_3 and 0.1 M H_2O_2. After this treatment, the electrode was reused for the new electrochemical uranium removal cycle. Ten consecutive cycles were performed under similar conditions.

Uranium Extraction from Natural Seawater

For purpose of examining the uranium extraction capacity of the PAF based electrode in natural seawater, the self-standing electrode was assembled in a flow device. The natural seawater without adjusting the pH value was forced through the electrode at a water flow of 5L

h^{-1}. Meanwhile, the voltage ranges from 0 V to −5 V using a frequency of 400 Hz were also applied to the electrodes. On specific days, the concentrations of residual uranium in the natural seawater were determined.

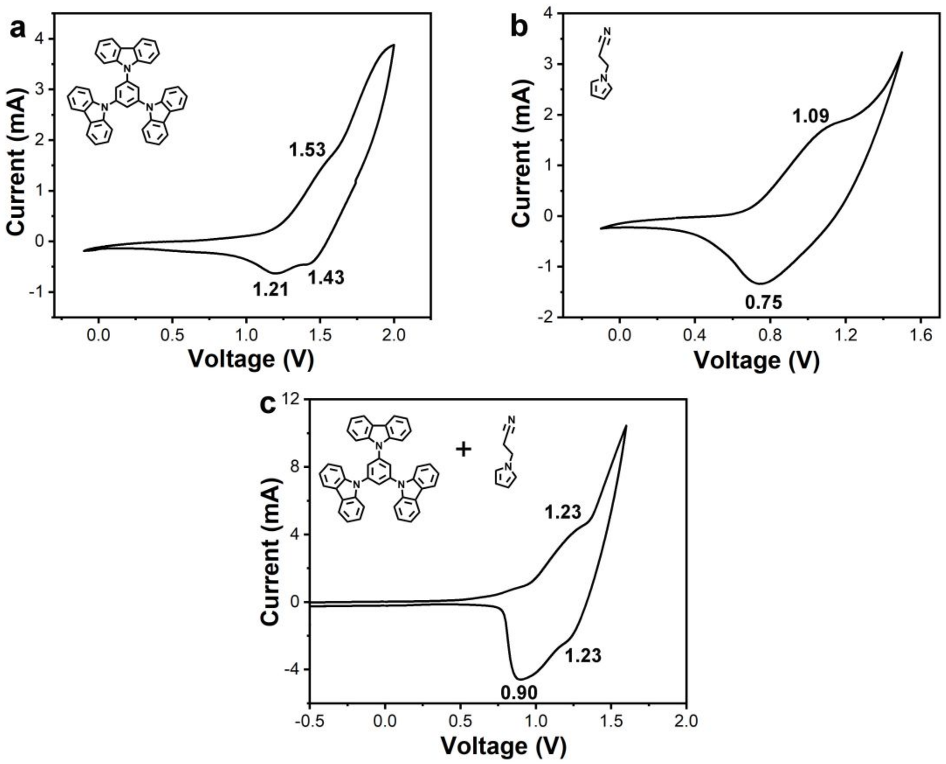

Figure S1. CV curves of the electropolymerization of only TCB (a), only NCP (b) and the coelectropolymerization of TCB and NCP (c).

Figures S1a and S1b show the current curves recorded in the first CV cycle with TCB and NCP, respectively, as the solo monomer. The oxidative peak potentials for TCB and NCP were 1.53 V and 1.09 V, respectively. There were two reduction peaks at 1.21 V and 1.43 V for the electropolymerization of TCB. One reduction peak at 0.75 V was observed for the electropolymerization of NCP. When using two kinds of monomers in the co-electropolymerization (Figures S1c), the oxidative and reduction potentials showed the shifts owing to the copolymerization process. The current curves indicated the success of co-electropolymerization of these two monomers.

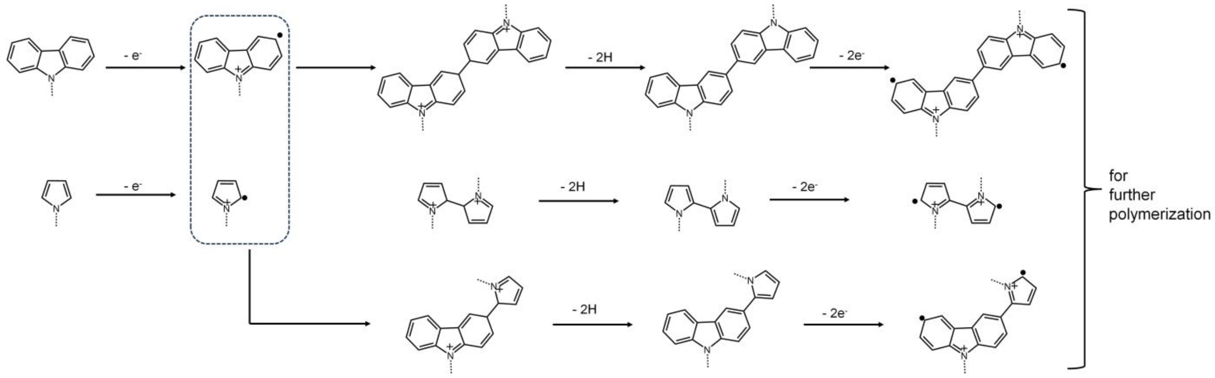

Figure S2. Mechanism of monomer oxidation, crosslinking, and reduction during the coelectropolymerization of TCB and NCP.

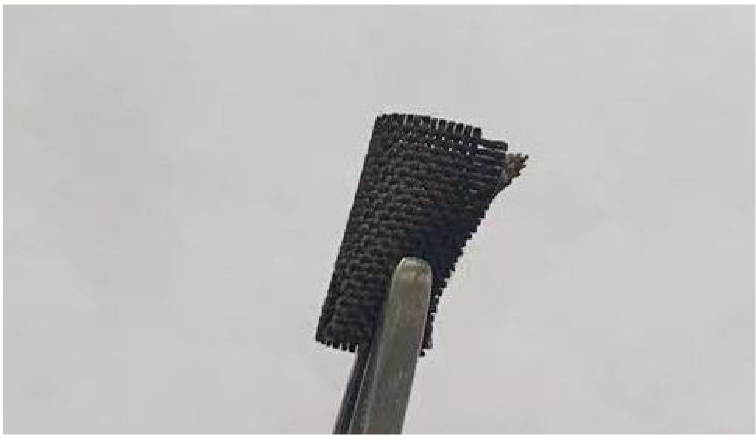

Figure S3. Optical image of the self-standing porous aromatic framework electrodes.

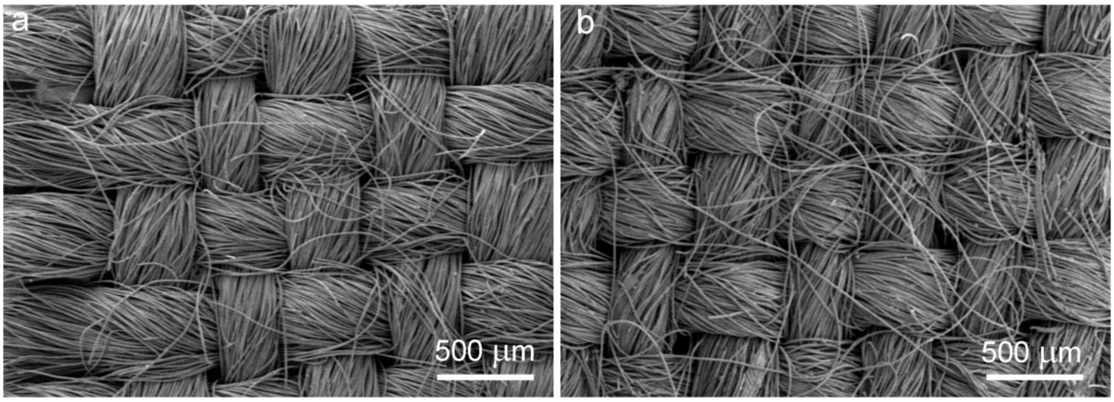

Figure S4. SEM images with low magnification for pure carbon cloth (a) and PAF-E (b).

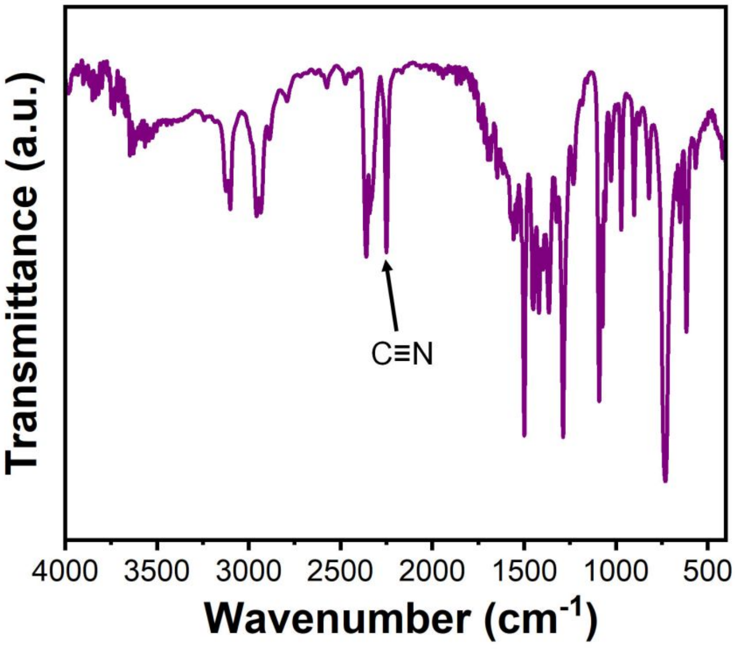

Figure S5. FT-IR spectrum of NCP.

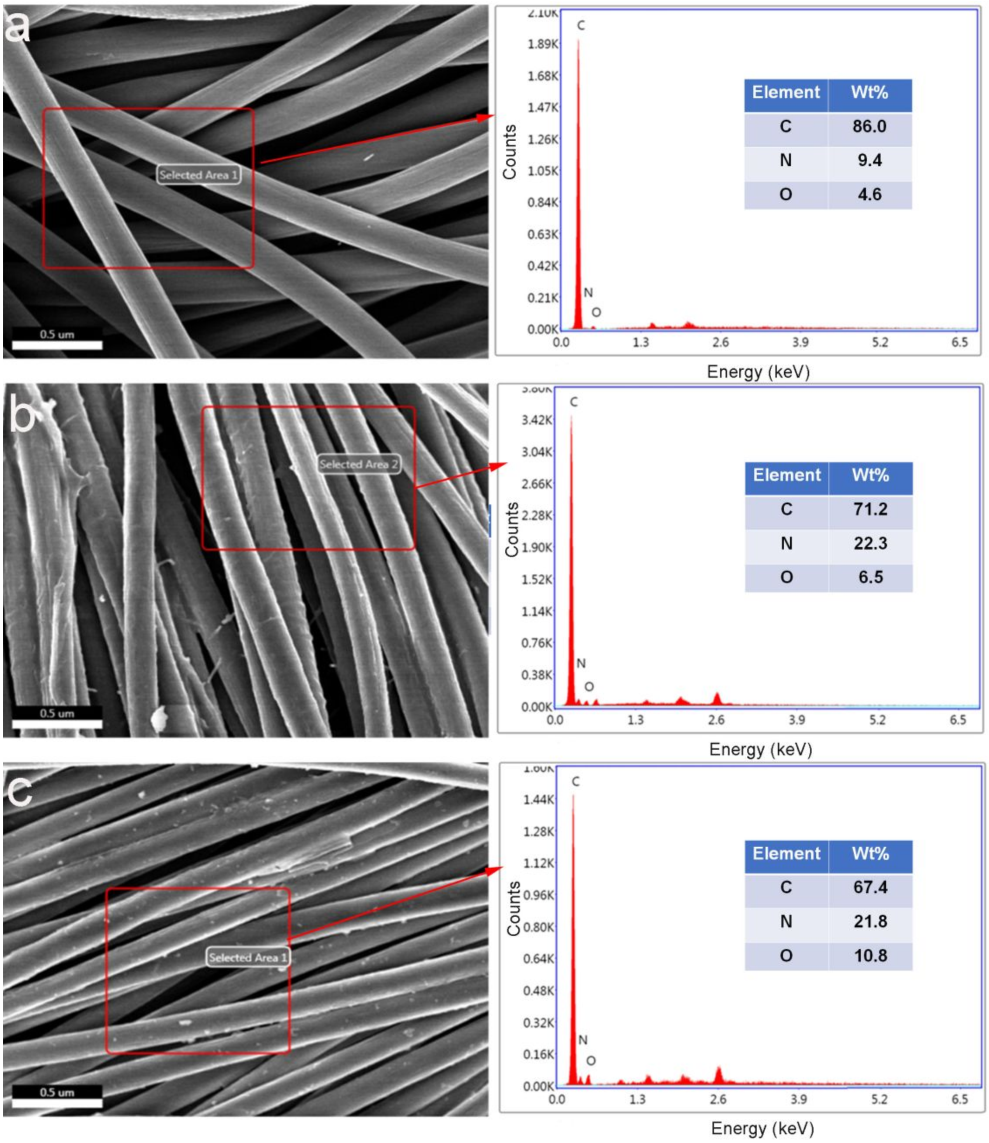

Figure S6. SEM-EDS patterns of carbon cloth (a), PAF-144@carbon cloth (b), and PAF-144-AO@carbon cloth (c).

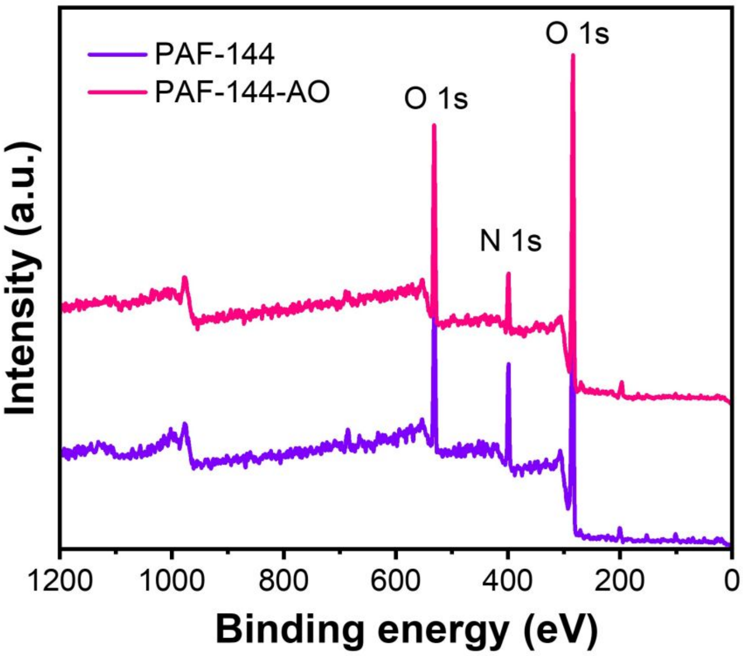

Figure S7. The survey XPS spectra of PAF-144 and PAF-144-AO.

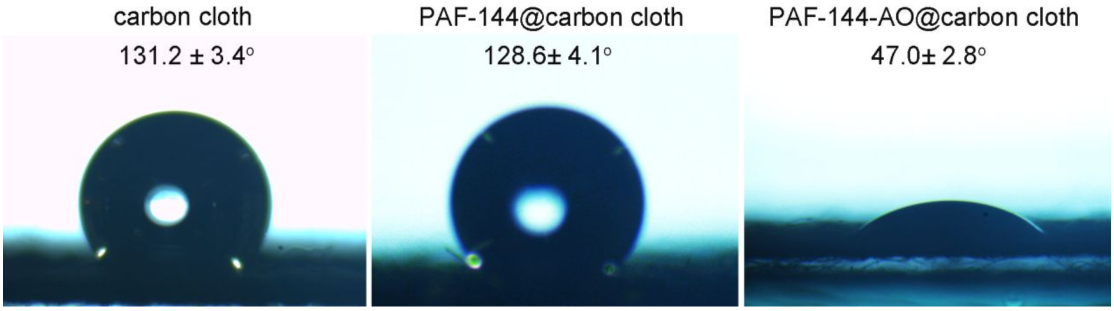

Figure S8. Water contact angles of obtained electrodes.

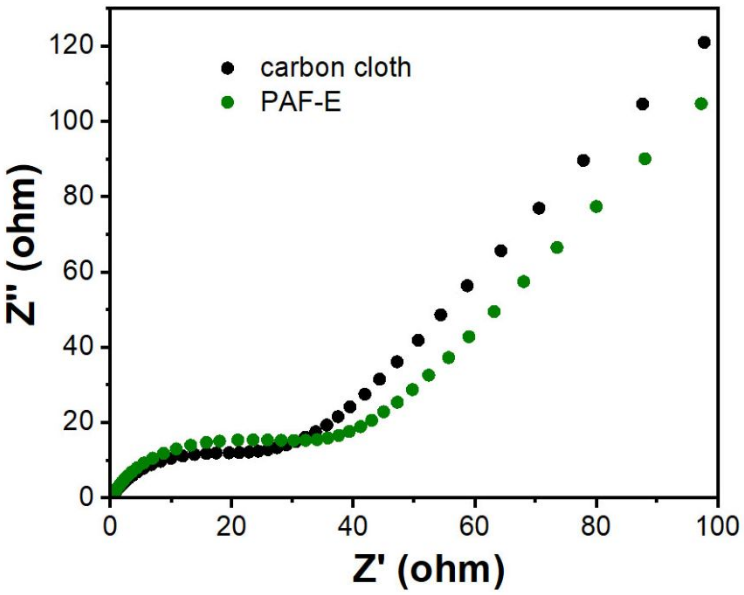

Figure S9. Electrochemical impedance Nyquist plots of pure carbon cloth and PAF-E.

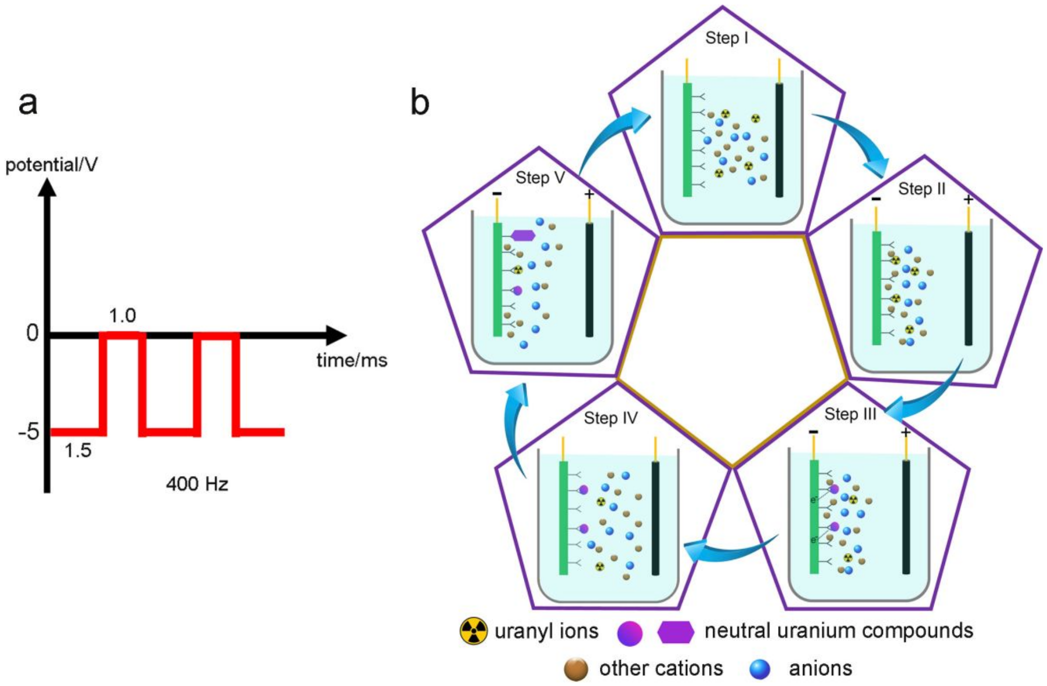

Figure S10. The voltage alternates based the HW-ACE method between −5 V and 0 V with a frequency of 400 Hz (a). Physical processes in the electrochemical extraction of uranium (b). In step I, all ions are dispersed in seawater solution in a random manner. In step II, under the action of the negative potential, uranyl ions start to migrate toward the PAF-E and are bound by the electrode. In step III, the adsorbed uranyl ions can be transformed into neutral U compounds which are deposited on the electrode surface. In step IV, when the potential becomes zero, other ions not bound with PAF-E will be released into the solution again. In step V, adsorption and electrocatalysis of uranyl ions continue and result in the larger growth of neutral U compounds.

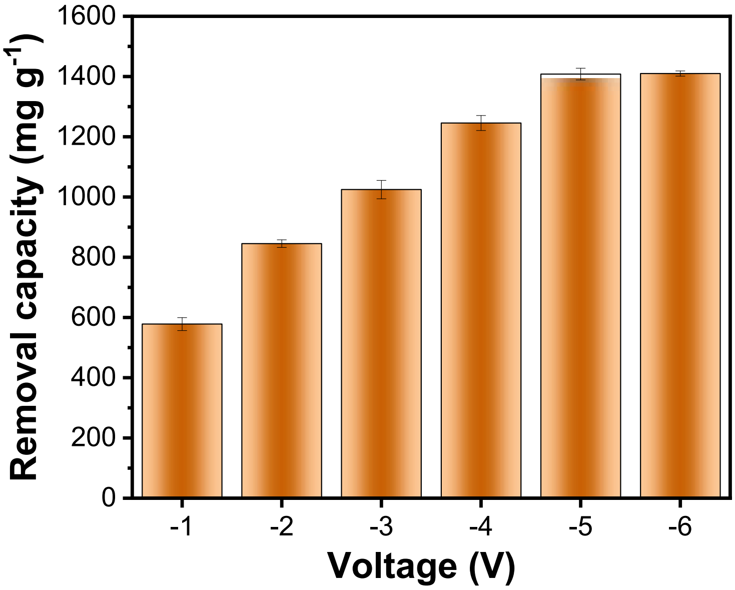

Figure S11. Effect of the applied voltage on the U removal using PAF-E (C_0 = 80 ppm).

The effect of the applied voltage on the uranium removal was investigated at different negative potentials from −1 V to −6 V. As the potential increased, the electrochemical uranium removal improved gradually and tended to the equilibrium after the potential of −5 V. High voltage could lead to the strong interaction between uranyl and PAF-E. Thus, the alternating voltage between −5 V and 0 V was applied in this work.

Table S1 Comparison of the uranium removal performance of different materials.

Materials	U removal capacity at low C_e[a]	U removal capacity at high C_e[b]	U extraction from natural seawater	Removal method	Ref.
PAF-144-AO	1413.9 mg g^{-1} (4.6 mg L^{-1})	7450.9 mg g^{-1}	12.6 mg g^{-1} (24 days)	electrochemical	This work
PA-PANI	205.6 mg g^{-1} (10.7 mg L^{-1})	256.0 mg g^{-1}	------	electrochemical	[1]
BC/PPy	120.3 mg g^{-1} (30.1 mg L^{-1})	237.9 mg g^{-1}	------	electrochemical	[2]
Fe–N_x–C–R	83.0 mg g^{-1} (16.0 mg L^{-1})	14302 mg g^{-1} [d]	1.2 mg g^{-1} (1 days)	electrochemical	[3]
CMM	158.9 mg g^{-1} (49.5 mg L^{-1})	582.4 mg g^{-1}	------	electrochemical	[4]
PPA@MISS-PAF-1	------[c]	307.0 mg g^{-1}	13.0 mg g^{-1} (56 days)	electrochemical	[5]
sp^2c-COF films	897.9 mg g^{-1} (34.1 mg L^{-1})	2475.0 mg g^{-1}	------	electrochemical	[6]
BSA@CFF	------	2850.0 mg g^{-1}	6.7 mg g^{-1} (18 days)	electrochemical	[7]
3D-FrGOF	610.6 mg g^{-1} (47.0 mg L^{-1})	4560.0 mg g^{-1}	------	electrochemical	[8]
TFPM-PDAN-AO	204.0 mg g^{-1} (38.1 mg L^{-1})	4685.0 mg g^{-1}	12.8 mg g^{-1} (20 days)	electrochemical	[9]
MSF@PAO-PEI	794.1 mg g^{-1} (40.7 mg L^{-1})	926.2 mg g^{-1}	1.1 mg g^{-1} (20 days)	physicochemical	[10]
COF-R_5	155.4 mg g^{-1} (4.8 mg L^{-1})	253.6 mg g^{-1}	11.3 mg g^{-1} (15 days)	physicochemical	[11]
Imprinted MOF	403.7 mg g^{-1} (7.7 mg L^{-1})	461.0 mg g^{-1}	7.4 mg g^{-1} (16 days)	physicochemical	[12]
ECP gels	407.9 mg g^{-1} (31.5 mg L^{-1})	970.2 mg g^{-1}	10.4 mg g^{-1} (28 days)	physicochemical	[13]
PAF-170-AO	469.1 mg g^{-1} (1.3 mg L^{-1})	702.0 mg g^{-1}	8.9 mg g^{-1} (60 days)	physicochemical	[14]
Fe@PDA-PAO	862.7 mg g^{-1} (15.7 mg L^{-1})	1086.0 mg g^{-1}	12.3 mg g^{-1} (48 days)	photothermal enhanced	[15]
GO-BPG-GLACS	532.0 mg g^{-1} (13.4 mg L^{-1})	662.0 mg g^{-1}	9.2 mg g^{-1} (14 days)	photothermal enhanced	[16]
COF 4-Pd-AO	135.4 mg g^{-1} (5.4 mg L^{-1})	155.7 mg g^{-1}	4.6 mg g^{-1} (1 days)	adsorption–photocatalysis	[17]

[a]U removal capactiy at low equilibrium concentration in the adsorption isotherm curve;

[b]U removal capactiy at high initial equilibrium concentration or maximum adsorption capacity from Langmuir isotherm model;

[c]Not reported;

[d]This removal capacity was obtained from the high initial U concentration of 1000 ppm.

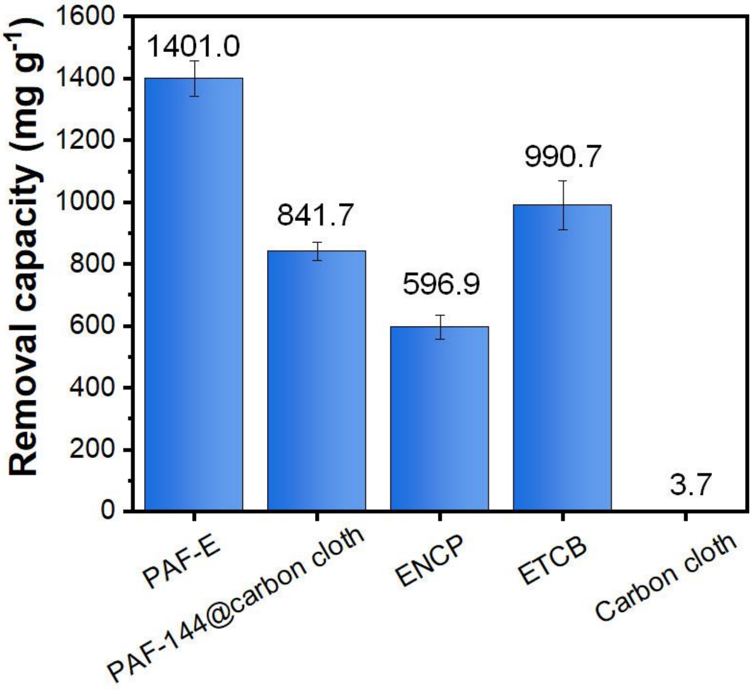

Figure S12. Uranium electrochemical removal by different electrodes (C_0 = 80 ppm).

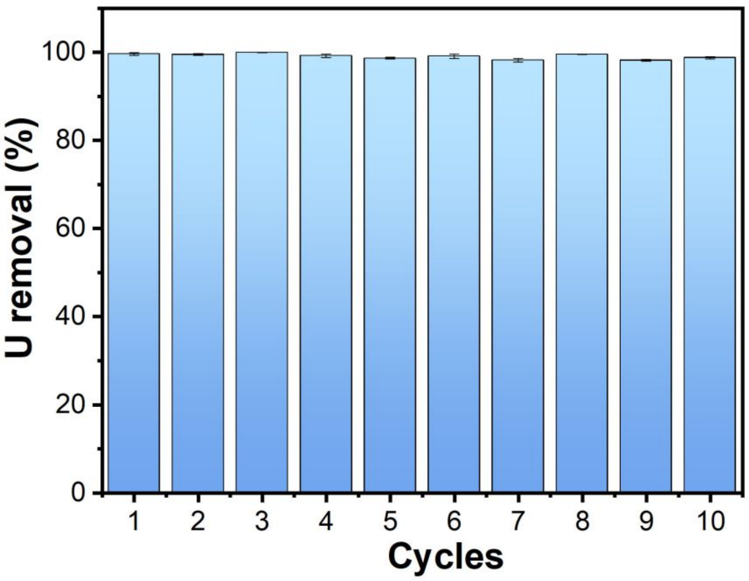

Figure S13. Durability performance of the PAP-E in 20 ppm uranium-spiked seawater.

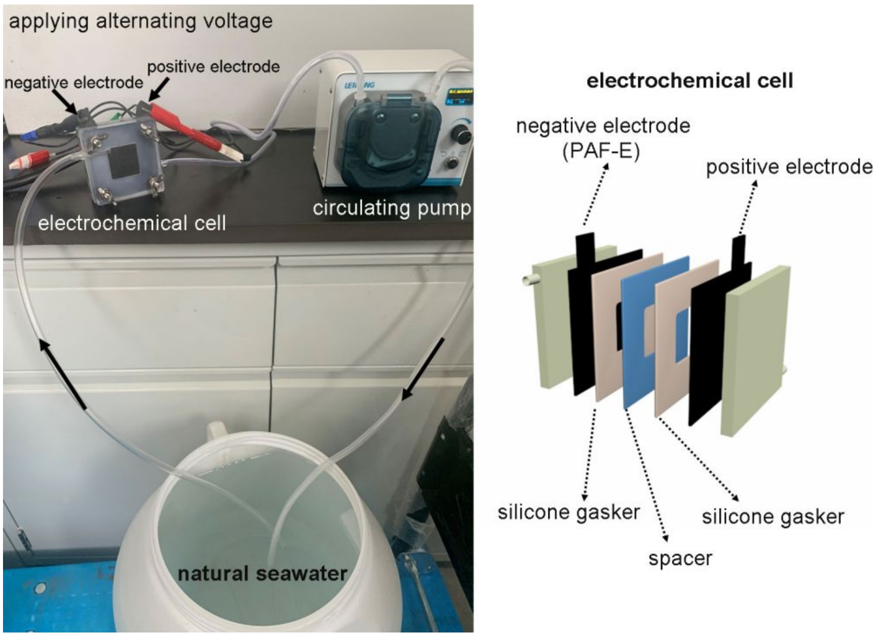

Figure S14. Device of the electrochemical uranium extraction from natural seawater.

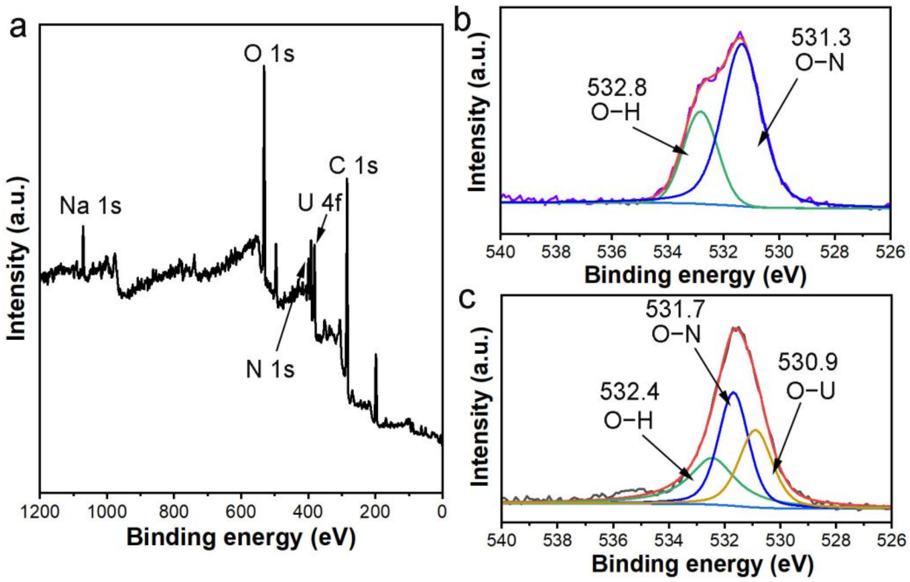

Figure S15. XPS survey spectrum of PAF-144-AO@ carbon cloth after the uranium extraction (a). High-resolution O 1s spectra of PAF-144-AO@ carbon cloth before (b) and after (c) after the uranium extraction.

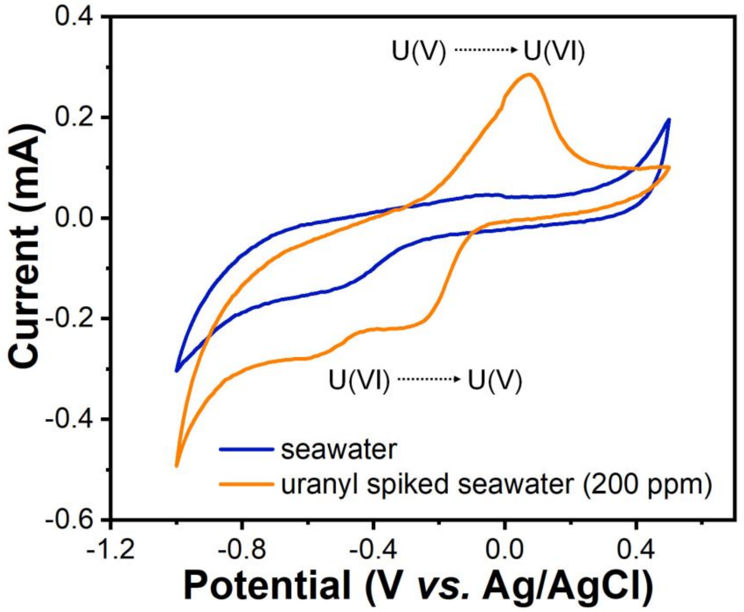

Figure S16. CV profiles of PAF-E in the natural seawater and uranium-spiked seawater (200 ppm).

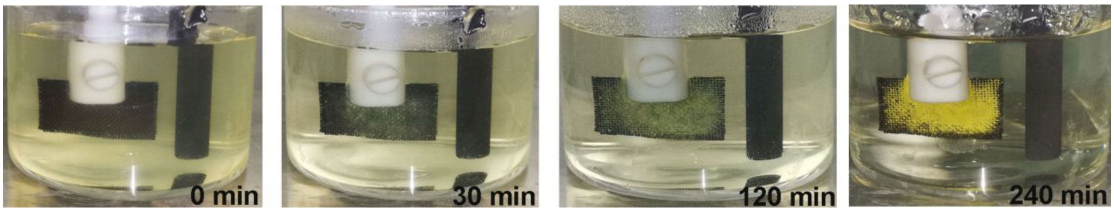

Figure S17. The formation process of yellow flocs on the PAF electrode during electrochemical uranium extraction (initial uranium concentration of ≈500 ppm).

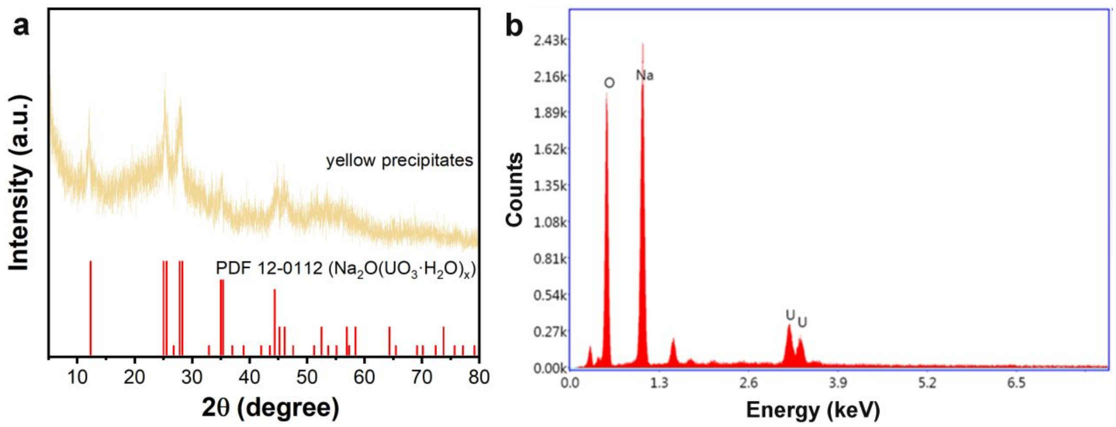

Figure S18. XRD (a) and EDS (b) pattern of the collected yellow precipitates.

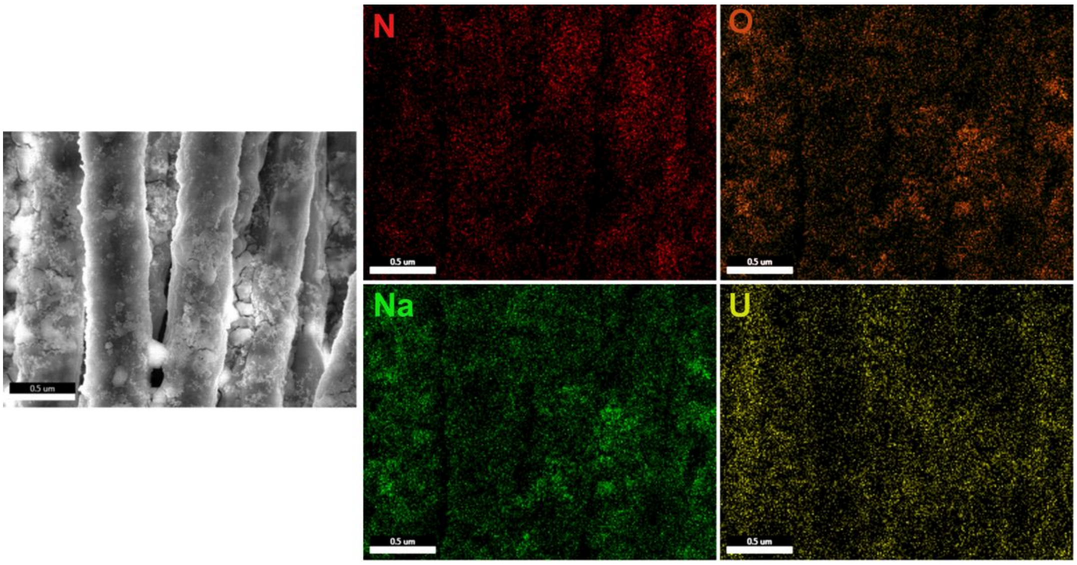

Figure S19. SEM image of the PAF-E after the electrochemical uranium extraction and its corresponding elemental mapping.

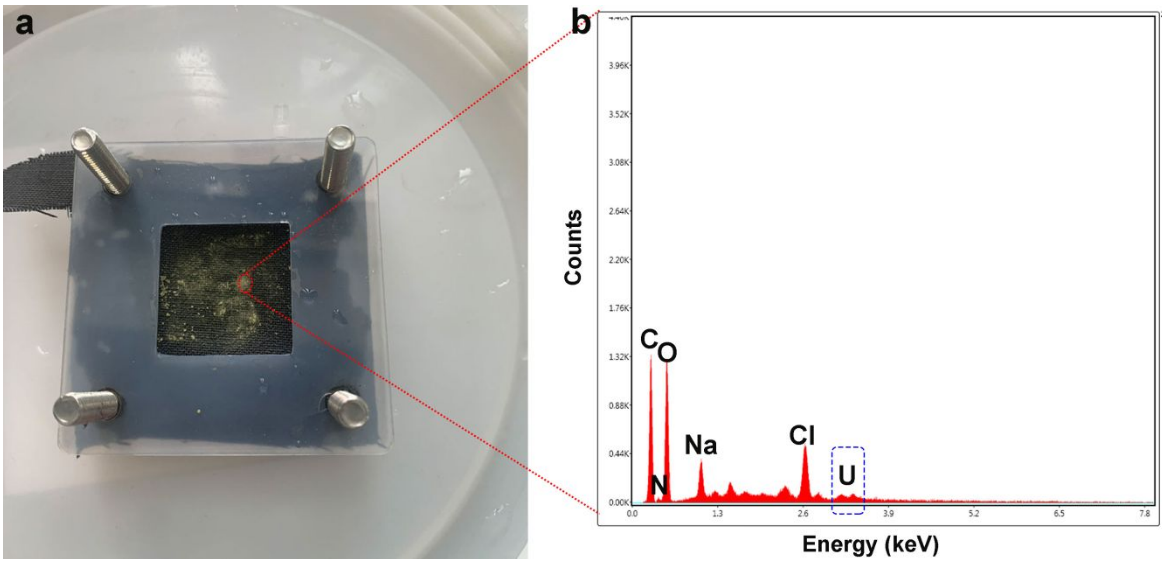

Figure S20. Optical picture of the PAF-E after the uranium extraction from the natural seawater (b) and its corresponding EDS spectrum reflecting the element composition.

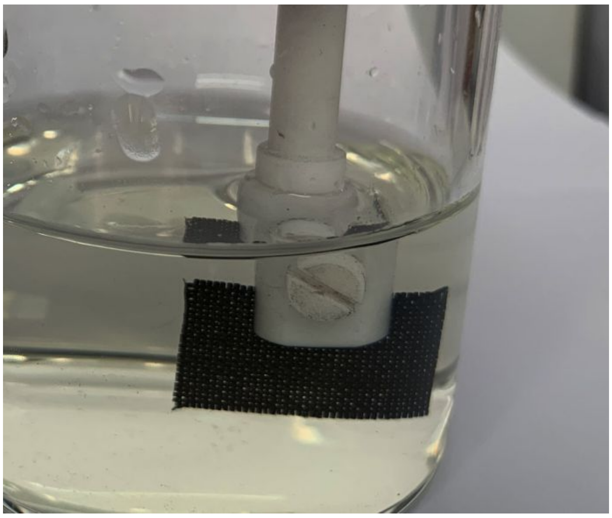

Figure S21. Optical picture of the electrochemical uranium extraction from U solution prepared using deionized water after 24 h (initial uranium concentration of ≈300 ppm).

References

1. Huang, M. N.; Xie, L. S.; Wang, Y. J.; He, H. J.; Yu, H. B.; Cui, J. C.; Feng, X. G.; Lou, Z. N.; Xiong, Y. Efficient uranium electrochemical deposition with a functional phytic Acid-Doped Polyaniline/Graphite sheet electrode by Adsorption-electrodeposition strategy. *Chem. Eng. J.* **2023**, *457*, 141221.

2. Yu, H. L.; Zhou, L. M.; Li, Z. Y.; Liu, Y. L.; Ao, X. Q.; Ouyang, J. B.; Le, Z. G.; Liu, Z. R.; Adesina, A. A. Electrodeposited polypyrrole/biomass-derived carbon composite electrodes with high hybrid capacitance and hierarchical porous structure for enhancing U(VI) electrosorption from aqueous solution. *Sep. Purif. Technol.* **2022**, *302*, 122169.

3. Yang, H.; Liu, X. L.; Hao, M. J.; Xie, H. Y.; Wang, X. K.; Tian, H.; Waterhouse, G. I. N.; Kruger, P. E.; Telfer, S. G.; Ma, S. Q. Functionalized iron-nitrogen-carbon electrocatalyst provides a reversible electron transfer platform for efficient uranium extraction from seawater. *Adv. Mater.* **2021**, *33*, 2106621.

4. Zhang, Y. Z.; Zhou, J.; Wang, D.; Cao, R. Y.; Li, J. X. Performance of MXene incorporated MOF-derived carbon electrode on deionization of uranium(VI). *Chem. Eng. J.* **2022**, *430*, 132702.

5. Wang, Z. Y.; Meng, Q. H.; Ma, R. C.; Wang, Z. K.; Yang, Y. J.; Sha, H. Y.; Ma, X. J.; Ruan, X. H.; Zou, X. Q.; Yuan, Y.; Zhu, G. S. Constructing an ion pathway for uranium extraction from seawater. *Chem* **2020**, *6*, 1683-1691.

6. Yan, H. K.; Kou, Z. H.; Li, S. X.; Zhang, T.; Synthesis of sp^2 carbon-conjugated covalent organic framework thin-films via copper-surface-mediated knoevenagel polycondensation. *Small* **2023**, *19*, 2207972.

7. Ye, H.; Li, T.-H.; Huang, Y.-Q.; Jin, J.-M.; Fei, J.-Y.; Wu, M.-B.; Yao, J. M. Amyloid-like coatings decorated electrodes boost the uranium electro-adsorption from seawater. *Chem. Eng. J.* **2023**, *451*, 138615.

8. Wang, C.; Helal, A. S.; Wang, Z. Q.; Zhou, J.; Yao, X. H.; Shi, Z.; Ren, Y.; Lee, J.; Chang, J.-K.; Fugetsu, B.; Li, J. Uranium in situ electrolytic deposition with a reusable functional graphene-foam electrode. *Adv. Mater.* **2021**, *33*, 2102633.

9. Zhang, C.-R.; Qi, J.-X.; Cui, W.-R.; Chen, X.-J.; Liu, X.; Yi, S.-M.; Niu, C.-P.; Liang, R.-P.; Qiu, J.-D. A novel 3D sp^2 carbon-linked covalent organic framework as a platform for efficient electro-extraction of uranium. *Sci. China Chem.* **2022**, *66*, 562-569.

10. Yang, J. J.; Li, Y.; Tian, T.; Shi, H. T.; Ahmad, Z.; Geng, N. B.; Jin, J.; Huang, Y. Q.; Zhang, H. J.; Fan, H. J.; Chen, J. P. Novel mesocellular silica foam supported poly(amidoxime-ethyleneimine) network for fast and highly efficient uranium extraction from seawater. *Chem. Eng. J.* **2023**, *465*, 142952.

11. Xie, Y. H.; Wu, Y.; Liu, X. L.; Hao, M. J.; Chen, Z. S.; Waterhouse, G. I. N.; Wang, X. K.; Yang, H.; Ma, S. Q.; Rational design of cooperative chelating sites on covalent organic frameworks for highly selective uranium extraction from seawater. *Cell Rep. Phys. Sci.* **2023**, *4*, 101220.

12. Feng, L. J.; Wang, H.; Feng, T. T.; Yan, B. J.; Yu, Q. H.; Zhang, J. C.; Guo, Z. H.; Yuan, Y. H.; Ma, C. X.; Liu, T.; Wang, N. In situ synthesis of uranyl-imprinted nanocage for selective uranium recovery from seawater. *Angew. Chem., Int. Ed.* **2022**, *61*, 82-86.

13. Pan, Z. H.; Zhao, L.; Cai, L. R.; Wang, S. Y.; Lu, B.; Zhang, P. Y.; Wang, G. Speeding up the selective extraction of uranium through in situ formed nano-pockets. *J. Mater. Chem. A* **2023**, *11*, 15437-15443.

14. Li, Z. N.; Meng, Q. H.; Yang, Y. J.; Zou, X. Q.; Yuan, Y.; Zhu, G. S. Constructing amidoxime-modified porous adsorbents with open architecture for cost-effective and efficient uranium extraction. *Chem. Sci.* **2020**, *11*, 4747-4752.

15. Liu, T.; Xie, Z. J.; Chen, M. W.; Tang, S.; Liu, Y. J.; Wang, J.; Zhang, R. Q.; Wang, H.; Guo, X.; Yuan, Y. H.; Wang, N. Mussel-inspired dual-crosslinked polyamidoxime

photothermal hydrogel with enhanced mechanical strength for highly efficient and selective uranium extraction from seawater. *Chem. Eng. J.* **2022**, *430*, 133182.

16. Li, T. Y.; Lin, X. B.; Zhang, Z. H.; Yang, L. S.; Qian, Y. C.; Fu, L.; Zhou, S. Y.; Chen, W. P.; Wang, Q. C.; Li, X.; Kong, X.-Y.; Xiao, H. Y.; Jiang, L.; Wen, L. P. Photothermal-enhanced uranium extraction from seawater: a biomass solar thermal collector with 3D ion-transport networks. *Adv. Funct. Mater.* **2023**, *33*, 2212819.

17. Hao, M. J.; Chen, Z. S.; Liu, X. L.; Liu, X. H.; Zhang, J. Y.; Yang, H.; Waterhouse, G. I. N.; Wang, X. K.; Ma, S. Q. Converging cooperative functions into the nanospace of covalent organic frameworks for efficient uranium extraction from seawater. *CCS Chemistry* **2022**, *4*, 2294-2307.
